# Prevalence of Iron Deficiency Anemia and Iron Deficiency Without Anemia Among Moderate and Severely Acute Malnourished Children

**DOI:** 10.7759/cureus.65633

**Published:** 2024-07-29

**Authors:** Suhas H S, Manoj Kumar, Tamanna Choudhary, Vikash Katewa, Pramod Sharma

**Affiliations:** 1 Pediatrics, Dr. S. N. Medical College, Jodhpur, IND

**Keywords:** s ferritin, severe acute malnutrition, moderate acute malnutrition, iron deficiency without anemia, iron deficiency anemia

## Abstract

Background: Iron deficiency remains one of the globally recognized leading causes of morbidity and mortality in children, among developing countries like India as compared to the Western world.

Objective: To estimate the prevalence of iron deficiency anemia (IDA) and iron deficiency without anemia (IDWA) among malnourished children.

Methods: This cross-sectional study was conducted in the Department Of Pediatrics, Tertiary Care Hospital, Western Rajasthan. Demographic data and serum samples were collected and analyzed. Hematological and biochemical values were determined for 300 children aged 6 months to 59 months.

Results: A total of 93.9% of severe acute malnutrition (SAM) children and 83.24% of moderate acute malnutrition (MAM) children had anemia as per WHO definition, with moderate anemia (47.66%) being the predominant type of anemia. About 64% of children showed iron deficiency with a prevalence of IDA and IDWA being 94.27% and 5.72%, respectively. The mean values of serum ferritin, serum iron, serum total iron binding capacity (TIBC), and transferrin saturation in children with IDWA were 8.34±2.85, 17.43±7.57, 454.09±40.76, and 4.09±1.44, respectively.

Conclusion: The proportion of anemic children in both SAM and MAM groups was very high. Our study shows that more than 60% of the MAM and SAM children were iron deficient. We recommend future measures for the prevention and control of anemia, including increased coverage of nutritional supplementation, fortification programs, and supplement iron in this sub-group (IDWA) to take care of their symptoms due to iron deficiency even before the development of overt IDA.

## Introduction

According to the World Health Organization (WHO), anemia is one of the 10 most serious health problems in the world [1]. India is one of the countries with the highest prevalence of anemia. As per NFHS-5 (2019-21), the highest spike in anemia is seen among children aged 6-59 months. A total of 67.1% of children were anemic as reported in NFHS-5 as against a total of 58.6% reported in NFHS-4 (2015-2016) and is still a major social and medical problem [2]. Iron deficiency anemia (IDA) is the most common cause of anemia and is commonest among malnourished children too. Iron deficiency adversely affects cognitive performance, behavior, physical growth of infants, immune status, and morbidity from infections [3].

Severe acute malnutrition (SAM) children with anemia have been shown to have higher mortality as compared to SAM children without anemia [4]. Even before the development of anemia in iron deficiency, individuals pass through different stages of iron deficiency such as pre-latent, latent, pre-anemic, and anemic [5]. Not many studies have been conducted to estimate the prevalence of iron deficiency without anemia (IDWA). Identifying and treating the iron-deficient erythropoietic stage even before the development of IDA would help to reduce morbidity and mortality. This study is planned to estimate the overall burden of malnourished children with IDA and IDWA.

## Materials and methods

A hospital-based cross-sectional observational study was conducted in the Tertiary Care Hospital, Rajasthan, from June 2022 to November 2022 after obtaining clearance from the Institutional Ethics Committee, Dr. S. N. Medical College, Jodhpur, India. A total of 300 children aged 6 months to 59 months who fulfilled the WHO definition of moderate acute malnutrition (MAM) (185) and SAM (115) attending OPD or admitted in the wards at Umaid Hospital, Jodhpur, India, or MDM Hospital, Jodhpur, India, were included in the study. Children with pre-existing congenital and chronic diseases that disrupt iron metabolism, like celiac disease and thalassemia, and those who have been on any iron therapy for more than 15 days continuously at any time in the past three months were excluded.

A complete detailed history was noted as mentioned in a predesigned proforma. The venous blood of the child was drawn under aseptic precautions after due consent. The sample was analyzed for complete hemogram, iron parameters like serum ferritin, total iron, transferrin saturation, and total iron binding capacity (TIBC) by using the SYSMEX XE-2100 Hematology Automated Analyzer (Sysmex, Kobe, Japan) and the Bechman and Coulter Access-2 Chemiluminescence Automatic Analyzer (Beckman Coulter, Inc., Brea, United States).

Definitions used for the study

Severe Acute Malnutrition

SAM child is defined by low weight-for-height/length (Z-score below -3 SD of the median WHO child growth standards), a mid-upper arm circumference <115 mm, or the presence of nutritional edema [6].

Moderate Acute Malnutrition

MAM child is one with 70-80% of median weight-for-height (Z-score of <-3 SD to <-2 SD) or a mid-upper arm circumference of 115-125 mm and no edema and the child should have an appetite, be alert and clinically well [6].

Anemia

WHO’s criterion for anemia in children aged 6 months to 59 months is hemoglobin (Hb) levels <11 g/dL [3].

The classification of anemia severity is as follows: mild anemia is defined as Hb levels between 10 and 10.9 g/dL, moderate anemia is defined as Hb levels between 7 and 9.9 g/dL, and severe anemia is defined as Hb levels <7 g/dL.

Iron Deficiency

Iron deficiency was defined following a multiple-index model, proposed by the Chinese Medical Association in 2010 [7], as the presence of two or more of the following four abnormal values: (i) serum ferritin <15 ng/ml; (ii) serum iron <10.7 μmol/l; (iii) TIBC >62.7 μmol/l; and (iv) transferrin saturation <15%.

Iron Deficiency Anemia

IDA is defined as iron-deficient erythropoiesis in which individual hemoglobin levels are below two standard deviations (-2 SD) of the distribution mean for hemoglobin in an otherwise normal population of the same gender and age who are living at the same altitude [3].

Iron Deficiency Without Anemia

IDWA is defined as an iron-deficient condition without affecting erythropoiesis to the extent of causing defined anemia [8].

Statistical analysis

Data were entered in Excel spreadsheets and analyzed using IBM SPSS Statistics for Windows, Version 21 (Released 2012; IBM Corp., Armonk, New York, United States). Data were expressed as a mean and standard deviation which followed a normal distribution. The data were graphically presented in the form of vertical bars, tables, horizontal bars, and pie diagrams. P-value < 0.05 was taken as significant. Data were analyzed using Epi info statistical software.

## Results

A total of 185 SAM children and 115 MAM children were enrolled in the study. The mean age of the study group was 20.72±10.21 months with the maximum number of patients being in the age group of 12-24 months (35.67%). The majority of children were female (59.33%); the maximum number of children were from rural areas (53.67%) and more Hindus (70.67%) were there in the study group. The baseline characteristics of children taken in the study based on the severity of anemia are shown in Table 1. About 47.66% show moderate anemia followed by mild anemia which was observed in 20.00%. The most common presenting complaints were loose stools (28.67%), difficulty in respiration (23.67%), generalized weakness (18.33%), and vomiting (11.00%). The mean weight, height/length, and mid-upper arm circumference (MUAC) were 7.86±1.54 kg, 77.12±8.37 cm, and 11.49±0.59 cm, respectively. The prevalence of anemia among SAM and MAM children was 93.9% and 83.24%, respectively, as per the WHO cut-off. Maximum children had microcytic hypochromic anemia (SAM: 73.04%, MAM: 58.92%) followed by macrocytic anemia (SAM: 13.04%, MAM: 16.22%). The overall mean values of serum ferritin, serum iron, serum TIBC, and transferrin saturation were 16.22±12.05 ng/ml, 40.49±19.41 μmol/l, 392.30±79.30 μmol/l, and 10.94±5.90%, respectively. There was a statistically significant difference in the mean value of serum ferritin, serum TIBC, and transferrin saturation when compared between MAM and SAM children with no anemia, respectively (p-value=0.013, p-value=0.029, p-value=0.025). Similarly, a statistically significant difference was observed in mean serum ferritin among children having severe anemia and no anemia when compared with SAM and MAM children, respectively (p-value=0.002, p-value=0.016) (Table 2).

**Table 1:** Baseline characteristics based on severity of anemia

Severity of anemia (n=300)	No anemia ≥11 (g/dl) (n=38)(12.66%)	Mild anemia 10-10.9 (g/dl) (n=60) (20%)	Moderate anemia 7-9.9 (g/dl) (n=143) (47.66%)	Severe anemia <7 (g/dl) (n=59)(19.66%)
Male (n=122)	17 (13.99%)	27 (22.13%)	49 (40.16%)	29 (23.77%)
Female (n=178)	21 (11.79%)	33 (18.53%)	94 (52.80%)	30 (16.85%)
Religion wise				
Hindu (n=212)	28 (13.20%)	50 (23.58%)	100 (47.16%)	34 (16.03%)
Muslim (n=88)	10 (11.36%)	10 (11.36%)	43 (48.86%)	25 (28.40%)
Residence wise				
Urban (n=139)	24 (17.26%)	23 (16.54%)	66 (47.48%)	26 (18.70%)
Rural (n=161)	14 (8.69%)	37 (22.98%)	77 (47.82%)	33 (20.49%)

**Table 2:** Distribution based on severity of anemia and iron parameters TIBC: total iron binding capacity; SAM: severe acute malnutrition; MAM: moderate acute malnutrition

Severity of anemia	Serum ferritin (mean±SD)			Serum iron (mean±SD)			Serum TIBC (mean±SD)			Serum transferrin saturation (mean±SD)		
	MAM	SAM	p-value	MAM	SAM	p-value	MAM	SAM	p-value	MAM	SAM	p-value
No anemia (n=38)	29.65±13.08	15.85±1.78	0.016	37.45±17.16	25.22±10.24	0.079	309.54±94.16	407.42±140.78	0.029	13.35±5.60	7.85±5.81	0.025
Mild anemia (n=60)	18.26±14.35	14.13±9.19	0.187	39.41±17.09	35.54 19.78	0.422	400.79±59.78	405.61±91.03	0.81	10.37±5.68	9.68±6.80	0.674
Moderate anemia (n=143)	14.10±7.55	16.89±17.33	0.181	42.75±21.34	46.48±18.76	0.319	405.56±60.48	387.25±76.40	0.126	10.76±5.89	12.65±5.60	0.074
Severe anemia (n=59)	15.59±9.21	9.77±4.61	0.002	42.98±17.74	35.43±17.54	0.108	417.50±57.72	394.00±78.49	0.206	10.53±4.72	9.60±6.04	0.521
Overall (n=300)	17.57±11.52	14.04±12.60	0.013	41.37±19.55	39.07±19.78	0.318	390.40±75.74	395.36±84.96	0.599	11.10±5.70	10.68±6.21	0.551

About 64% of children showed iron deficiency with the prevalence of IDA and IDWA being 94.27% and 5.72%, respectively. The mean values of serum ferritin, serum iron, serum TIBC, and transferrin saturation of iron deficiency without anemic children were 8.34±2.85 ng/ml, 17.43±7.57 μmol/l, 454.09±40.76 μmol/l, and 4.09±1.44%, respectively. There were statistically significant differences in the mean value of serum iron and serum transferrin saturation observed when compared between IDA and IDWA among MAM children, respectively (p-value<0.0001, p-value<0.0001) (Table 3). There was a statistically significant positive correlation observed between hemoglobin levels (MAM) and serum ferritin (r=0.38, p≤0.001), and a statistically significant negative correlation was observed between hemoglobin (MAM) levels and serum TIBC (r=0.38, p≤0.001).

**Table 3:** Distribution of iron profile based on IDA status and severity of malnutrition TIBC: total iron binding capacity; SAM: severe acute malnutrition; MAM: moderate acute malnutrition; IDA: iron deficiency anemia; IDWA: iron deficiency without anemia

Iron deficiency anemia	Serum ferritin (mean±SD)			Serum iron (mean±SD)			Serum TIBC (mean±SD)			Serum transferrin saturation (mean±SD)		
	MAM	SAM	p-value	MAM	SAM	p-value	MAM	SAM	p-value	MAM	SAM	p-value
Present (IDA) (n=181)	10.06±3.12	9.64±3.44	0.388	33.15±9.99	32.23±11.56	0.571	419.59±53.20	417.03±70.59	0.781	7.87±2.42	8.00±3.09	0.758
Absent (IDWA) (n=11)	9.32±2.25	6.62±3.30	0.137	15.37±2.50	21.05±12.30	0.251	437.00±30.21	484.00±42.88	0.060	3.85±0.69	4.50±2.38	0.507
p-value	0.539	0.090	-	<0.0001	0.063	-	0.394	0.064	-	<0.0001	0.058	-

## Discussion

Anemia is still a major social and medical problem among children between 6 and 59 months of age [9]. Microcytic anemia is the most common cause of anemia, if not diagnosed and treated may become severe which may lead to pediatric morbidity, hospitalization, and mortality [10]. It has been seen that SAM with anemia shows higher mortality as compared to children without anemia [9]. We included SAM and MAM children in the 6-month to 59-month age group and found that there were 59.33% females and 40.67% males. The mean age of the children in our study was 20.72±10.21 months. A majority (35.67%) of children were between 12 months to 24 months of age. In a study by Jangid et al., the male-to-female ratio was 1.30:1 and the mean age of the children was 23±13.83 months [10]. Dwivedi et al. reported 100 cases of SAM, of which 62% were males and 38% were females [9]. The mean age of the children was 15.85 months.

In the current study, 53.67% were from rural dwellings. Similar to the current study anemia was found higher among rural residents than urban residents by Jondhale et al. and Tesfaye et al., respectively [11-12]. In our study, we found that the majority of the children had moderate anemia (47.66%), mild anemia was seen in 20% of children, severe anemia was observed in 19.66% of children, and 12.66% of children were not having anemia. In the study by Simbauranga et al., 33% had moderate anemia, 27.7% of children had severe anemia, and 16.5% had mild anemia [13]. In a study by Dwivedi et al., the majority of SAM children had moderate anemia (42%) with mild anemia in 24% and severe anemia in only 19% of patients, and 15% of children were without anemia [9]. In contrast, Thakur et al. found that a majority (67.3%) had severe anemia and 13.8% had moderate anemia [14].

In the current study out of 115 severely acute malnourished children, 93.9% of children had anemia and 6.08% were without anemia. Among 185 moderate acute malnourished children, 83.24% of children had anemia and 16.75% were without anemia. In our study among severely acute malnourished children, 73.04% had microcytic hypochromic anemia and 13.04% had macrocytic anemia. Similarly among moderately acute malnourished children, 58.92% had microcytic hypochromic anemia and 16.22% had macrocytic anemia. In another study by Jangid et al., 55.06% of the study group had microcytic anemia followed by dimorphic in 37.65% and macrocytic anemia in 5.26% [10]. In another study by Thakur et al., 38.6% had microcytic anemia followed by 30.5% having megaloblastic anemia [14]. Our study group had mean values of serum ferritin, serum iron, serum TIBC, and transferrin saturation 16.22±12.05, 40.49±19.41, 392.30±79.30, and 10.94±5.90, respectively. With IQR being 20.67-9.12, 49.0-27.00, 444.0-347.0, and 15.22-6.00, respectively. In another study conducted by Jangid et al., the study group had a mean serum ferritin level of 14.16±14.87 and IQR being 8.4 (6.300-12.300) [10].

In our study, 64% (n=192) showed an iron-deficient state. Out of which prevalence of IDWA and IDA were 5.72% (n=11) and 94.27% (181), respectively. While 36% (n=108) did not show an iron-deficient state. Among these children without iron deficiency, 75% (n=81) were having anemia other than IDA. Similarly in another epidemiological study by Zhu et al., the prevalence rates of iron depletion (ID) and IDA were 32.5% and 7.8%, respectively [15]. In another study conducted by Kessy et al. prevalence of ID, iron deficiency with no anemia, and IDA were 2.6% (n=8), 9.6% (n=29), and 28.1% (n=84) respectively [16].

The major limitation of our study is that we did not consider an apparently healthy population and serum levels of vitamin B12 and folic acid were not done. We recommend more studies to identify and treat IDWA and to reduce the morbidity and mortality in this subgroup of patients.

## Conclusions

This study emphasizes the fact that the proportion of anemia in both SAM and MAM children was very high. Various public health programs are running for these children which are aimed at iron supplementation but their impact is not percolating up to the beneficiary. We recommend further measures for the prevention and control of anemia, including increased coverage of nutritional supplementation and fortification programs. A high percentage of dimorphic and macrocytic anemia in malnourished children points to the need for folic acid and B12 supplementation in recommended dosages. IDWA needs a close look as the supplementation of iron in this subgroup would take care of their symptoms due to iron deficiency even if these children do not fall under the classical WHO definition of anemia.
